# Fire Analysis of Timber-Framed Walls Lined with Gypsum

**DOI:** 10.3390/ma15030741

**Published:** 2022-01-19

**Authors:** Paulo A. G. Piloto, Sergio Rodríguez-del-Río, Diego Vergara

**Affiliations:** 1 Departamento de Mecânica Aplicada, Campus de Santa Apolónia, Instituto Politécnico de Bragança, 5300-253 Bragança, Portugal; ppiloto@ipb.pt; 2 Department of Mechanical Engineering, Catholic University of Ávila, C/Canteros, s/n, 05005 Avila, Spain; sergiorodriguezdelrio@hotmail.com

**Keywords:** fire resistance, timber-framed walls, gypsum plasterboard

## Abstract

This investigation analyses the influence of the depth and the distance between studs on the fire resistance of lightweight timber-framed (LTF) walls lined with gypsum plasterboards. The simplified model used to determine the fire resistance in Eurocode EN 1995-1-2 provides very conservative values, as few parameters are considered. The new generation of Eurocode EN 1995-1-2 includes an upgrade of the simplified model, allowing us to predict the fire resistance of LTF wall assemblies more accurately. This separating function method considers the number, the thickness and the material of the protection layers, but does not explicitly consider the variation of the depth and the distance between the studs for the calculation of the insulation time of the assembly, besides including some limitations for both parameters. To demonstrate the influence of these parameters, 36 numerical simulations were carried out using the finite element method previously validated with experimental tests. The results obtained from the parametric analyses confirmed that such parameters affect the fire resistance of the LTF wall assemblies in a significant way. In addition, the results revealed an important contribution in the study of LTF wall assemblies against fire resistance, demonstrating the need for including extra geometric parameters in the simplified model in order to increase the accuracy of current models.

## 1. Introduction

Lightweight timber-framed (LTF) walls are commonly used in residential buildings due to their light weight and low construction costs. LTF walls are made with solid wood members (studs and tracks) used on buildings, for load-bearing and partition walls. The cladding for internal walls may be developed by gypsum panels (layers). The number of protection layers and insulation materials used in the cavities of the wall depends on the thermal and acoustic efficiency required to the LTF structures at room temperature, but also depends on the required fire rating of LTF walls. During recent years, several experimental tests have been carried out on LFF walls, which can be used for validation.

In 1996, Thomas [1] developed a one-dimensional heat-transfer model, in addition to performing a comparison of noncharred areas. In 1998, Takeda and Mehaffey [2] developed major revisions in the model, improving the description of heat transfer through the two-dimensional computational model WALL2D. In 2001, Clancy [3] reviewed the progress made in modelling heat transfer through LTF structures exposed to fire.

The separating function method is based on the Component Additive Method, firstly developed in 2009 by Schleifer [4]. The total fire resistance of the insulation is determined by the sum of the protection layers considering different heat-transfer paths. The coefficients of the design method (basic values, correction time and position coefficients) are only applied to non-load-bearing LTF walls.

In 2010, based on physical models and numerical simulations for heat transfer through separating multiple layered constructions, Frangi et al. [5] also presented the simplified model for the verification of LTF wall structures. This method is already included in the new generation of the EN 1995-1-2 [6] and significantly improves the design method in the current version of EN 1995-1-2 [7]. This method is based on a component additive model, in which the fire resistance is obtained from the sum of resistance values obtained in each layer (protection, cavity, and insulation). The method has been well-presented for light timber-frame walls by Mäger et al. in 2017 [8] and by Mäger et al. in 2018 [9]. The procedure to implement new materials is also presented by Mäger et al. in 2019 [10]. The separating function method assumes that, with minimal requirements on detailing, integrity is satisfied when insulation criteria is satisfied.

According to Michael Rauch et al. in 2018 [11], the separating function method is applicable up to 60 min of fire resistance. Extension to 90 and 120 min is also possible with some restrictions. Void cavities and multilayer claddings may lead to very conservative results.

In 2016, Xu et al. [12] developed a comparative experimental study to investigate the effects of using three different fire-protection measures to improve the fire resistance of timber floor assemblies. This investigation highlighted the existence of a major weakness in the simplified model presented by the current version of the EN 1995-1-2 [7].

In 2020, Piloto and Fonseca [13] developed a numerical model to perform a parametric analysis regarding the fire resistance of LTF walls lined with gypsum plasterboards. The new simplified method, presented by Frangi et al. [5] looks to be overpredicting the fire resistance of the insulation when using one gypsum layer, and underpredicting the fire resistance when using two gypsum layers. The residual area for every standard-rated LTF wall depends on the size of the wood studs and depends on the level of protection layers.

**Figure 1:**
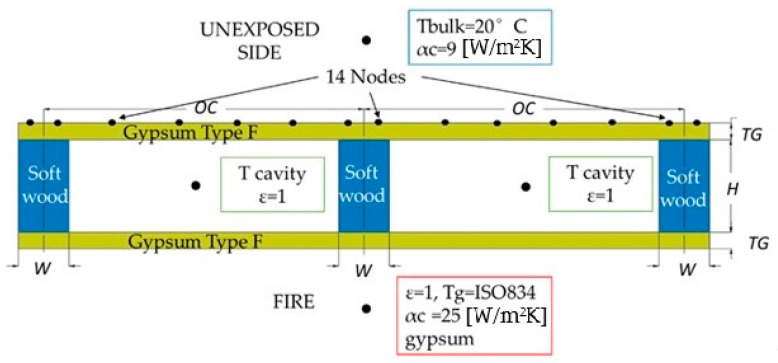
Geometric model and boundary conditions.

This investigation addresses how (i) the variation in the depth of the studs (*H*) and (ii) the variation in the stud spacing between centres (*OC*) affect the fire resistance of LTF walls’ insulation. In addition, this investigation determines the behaviour of the noncharred area of the studs, depending on *H* and depending on *OC* dimensions (Figure 1).

Thus, this paper deals with the fire effect on the non-load-bearing LTF wall structure cladded with gypsum plasterboard. To ensure the fire resistance of load-bearing walls, the criteria of the mechanical resistance (R), integrity (E) and insulation (I) must be verified, while the (E) and (I) criteria may be applied to non-load-bearing walls [7]. Furthermore, the simplified method presented by Frangi et al. [5], reported in [6], allows for obtaining the fire-resistance time of insulation of this type of wall, but more investigation is still required.

This investigation extends the contribution provided by Piloto and Fonseca [13] on the fire-resistance time of LTF walls by comparing it with the simplified model presented by Frangi et al. [5]. For this purpose, a two-dimensional parametric analysis of 36 different geometries based on a finite element model (FEM) is proposed, which is further validated with the experimental test of Takeda and Mehaffey [2]. This calculation only deals with the insulation’s fire resistance of the LTF wall.

## 2. Materials and Methods

The numerical model considers the cross-section of the structure, considering perfect contact between the materials. The boundary conditions applied for (i) the exposed side and (ii) the unexposed side are those of Eurocode EN 1991-1-2 [14]. On the exposed side, radiation with a fire emissivity coefficient (ε) value of 1 and a convection coefficient (αc) of 25 W/m^2^K is considered, using the standard ISO 834 curve as the bulk temperature. On the unexposed side, the simplified version for the boundary condition includes the radiation effect, using only the convection coefficient α_c_ = 9 W/m^2^K (Figure 1) [14]. An additional boundary condition is considered for the cavity region, considering only the radiation effect, performing the calculation of the cavity bulk temperature, and using the emissivity (ε) value of 1. The initial temperature of the LTF is constant and equal to T0 = 20 [°C] applied to all nodes of the model.

The finite element method is used to solve the nonlinear transient thermal analysis, considering the nonlinear behaviour of the thermal properties and an incremental solution process based on a variable time step (1 to 60 s). The nonlinear convergence criterion is based on the heat flow, considering a tolerance value of 0.001 and a reference value of 10^−6^ W. The 2D heat conduction inside the physical domain is mathematically modelled by the energy conservation equation (Equation (1)), where *T* represents the temperature [°C], *ρ*(*T*) is the specific mass [kg/m^3^], *C*_p_(*T*) is the specific heat [J/kgK], *λ*(*T*) is the thermal conductivity [W/mK], *t* is the time [s] and ∇ = (*∂x*, *∂y*) is the gradient. Equation (1) is based on the heat-flow balance for the infinitesimal material volume in each spatial direction.

(1) $$\rho \left(T\right)C_{p}\left(T\right)\frac{\partial T}{\partial t}=\nabla \cdot \left(\lambda \left(T\right)\nabla T\right)$$

Equation (1) is time-dependent because the heat flux on the boundary exposed to the fire change with time, i.e., the thermal state of the LTF is transient. The solution of Equation (1) is required to determine the temperature inside the physical domain over time, and consequently, to determine the fire char layer near the position of the 300 °C isothermal to define the moving of the pyrolysis process expected in wood.

The general procedure of the finite element method for solving Equation (1) is based on the weak-form Galerkin model and from the minimum condition for the weighted residual method, leading to the matrix format of the energy equation (Equation (2)):

(2) $$C_{\left(T^{n+1}\right)}\frac{T^{n+1}-T^{n}}{\Delta t}+\theta .K_{\left(T^{n+1}\right)}T^{n+1}=F_{\left(T^{n+1}\right)}-\left(1-\theta \right)K_{\left(T^{n+1}\right)}T^{n}$$

where the matrix $C_{\left(T^{n+1}\right)}$ is the capacitance matrix, *T^n^*˙ is the nodal vector for temperature at the time instant *t*_n_, $K_{\left(T^{n+1}\right)}$ is the conductivity matrix, and $F_{\left(T^{n+1}\right)}$ is the vector of the thermal load. 

**Figure 2:**
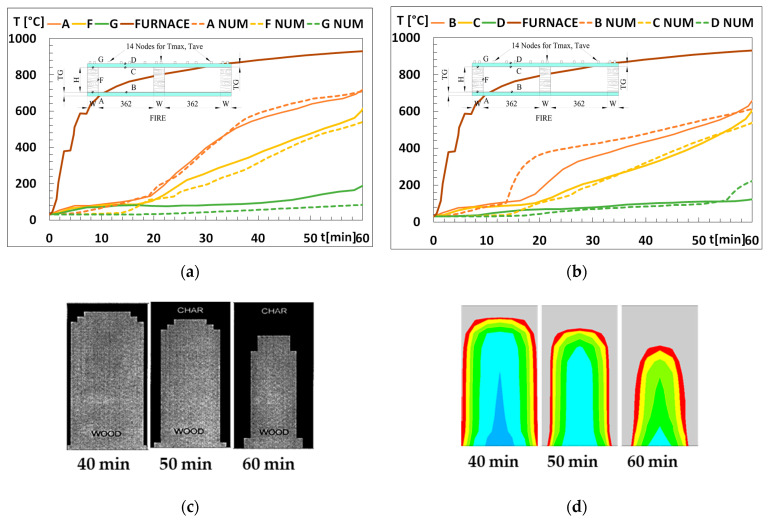
(**a**) Comparison of the temperature evolution at the stud’s points; (**b**) Comparison of the temperature evolution at gypsum plasterboards points; (**c**) Evolution of residual stud area [2]; (**d**) Evolution of residual stud area as numerical model used.

The following elements have been used to develop the numerical model: *PLANE55* and *SURF151*. The *SURF151* finite element provides the possibility to model the radiation in the cavity without considering the thermal degradation of the exposed gypsum layer. The mesh size has been determined by a convergence test of the solution. The number of elements increases with the distance *OC*, *H* and the thickness of gypsum layer (*TG*). The numerical model has been validated using the temperature in several points from experimental results provided by Takeda and Mehaffey [2], which also have allowed the validation of the noncharred area of the studs, Figure 2. The geometry for this validation model considers the stud depth *H* = 89 mm and width *W* = 38 mm, the thickness of the gypsum plate *TG* = 16 mm, and the distance between studs *OC* = 400 mm. The times used to validate the noncharred area are 40, 50 and 60 min. The boundary conditions are the ones defined in [2]. The numerical results are very close to the experimental results, so one can conclude that the model can extrapolate the experimental results to other dimensions.

For the parametric study, 36 simulations were carried out to determine the fire resistance and residual area after subjecting the walls to 30, 40, 50, and 60 min of fire exposure. The geometry of the structure is based on a wall formed by three studs and protected by a layer of gypsum plasterboard on each side. The value of *W* is considered constant in all cases, assuming *W* = 45 mm as it is the commonly used dimension [5]. The other dimensions are the following: *H* = 70, 90, and 130 mm; *TG* = 9.5, 12.5, and 15 mm; *OC* = 200, 300, 400, and 600 mm. The noncharred area of the central stud is selected for the assessment of the residual area. The fire curve used in the parametric analysis is the standard ISO-834 curve [15] and the thermal properties used for the materials are Softwood and Gypsum Type F, as defined by the new generation of EN 1995-1-2 [6].

The insulation’s fire resistance (I) is based on the readings of 14 nodal temperatures on the unexposed side of the wall (Figure 1), which are used to determine the maximum temperature (*T_max_*) and the average temperature (*T_ave_*) [13].

## 3. Results

The parametric analysis is developed to verify the effect of the size of the studs (*H*), the distance between them (*OC*), and the thickness of the gypsum layer. The results of the 36 simulations (Table 1) include the fire resistance (*T_ave_* and *T_max_*), considering the insulation criterion (I) of selecting the shortest time (*tins* (num)) at which the unexposed side reaches an increase of Δ*T_ave_* = 140 °C or Δ*T_max_* = 180 °C [7] above the initial average temperature of the nodes (20 °C). This result is compared with the time determined by the separating function method (*tins* (sim)).

Table 1 also shows the percentage of the noncharred area of the central stud, based on the 300 °C isothermal criterion. The results are determined by digital image processing. The values of *H*, *OC*, and *TG* influence the residual area of the central stud. This fact is very important for the calculation of the residual load-bearing capacity of any LTF wall structure affected by fire [6]. On average, the increase in fire resistance of the insulation is 5 ± 0.4 min for each millimetre of gypsum thickness. This result is only valid for the current used values of gypsum layer.

**Figure 3:**
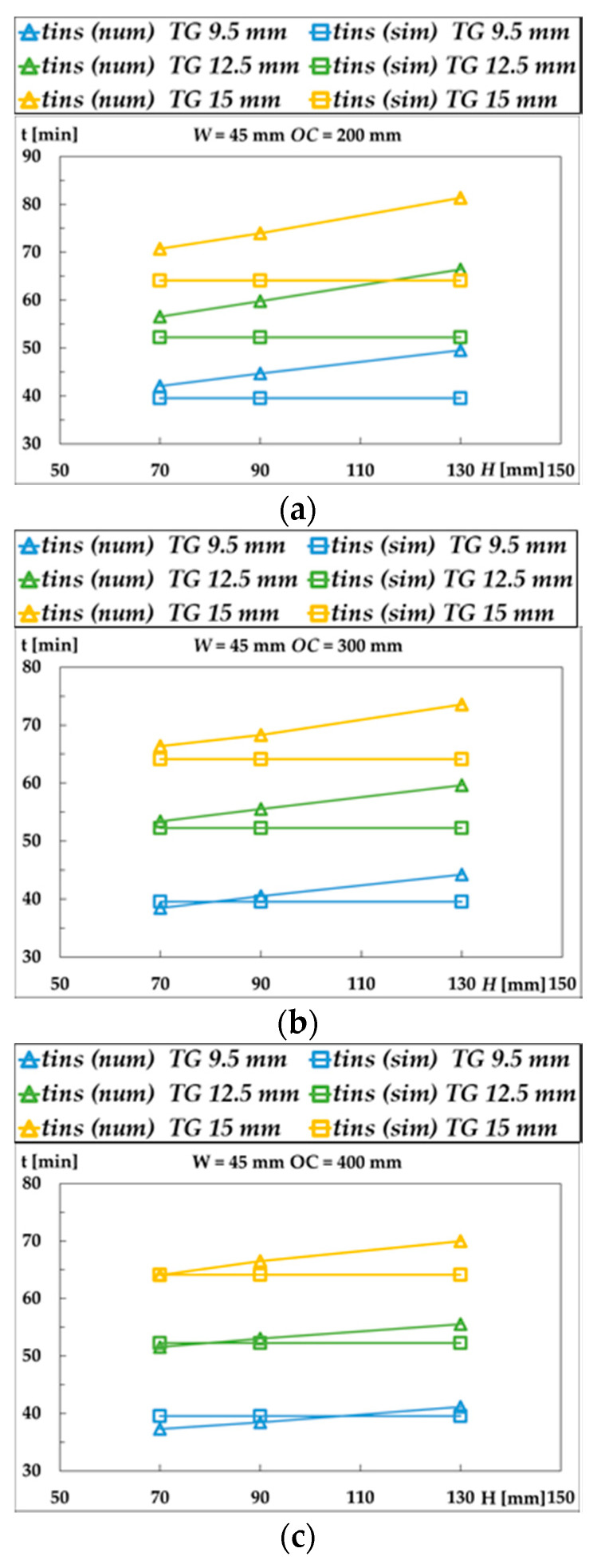

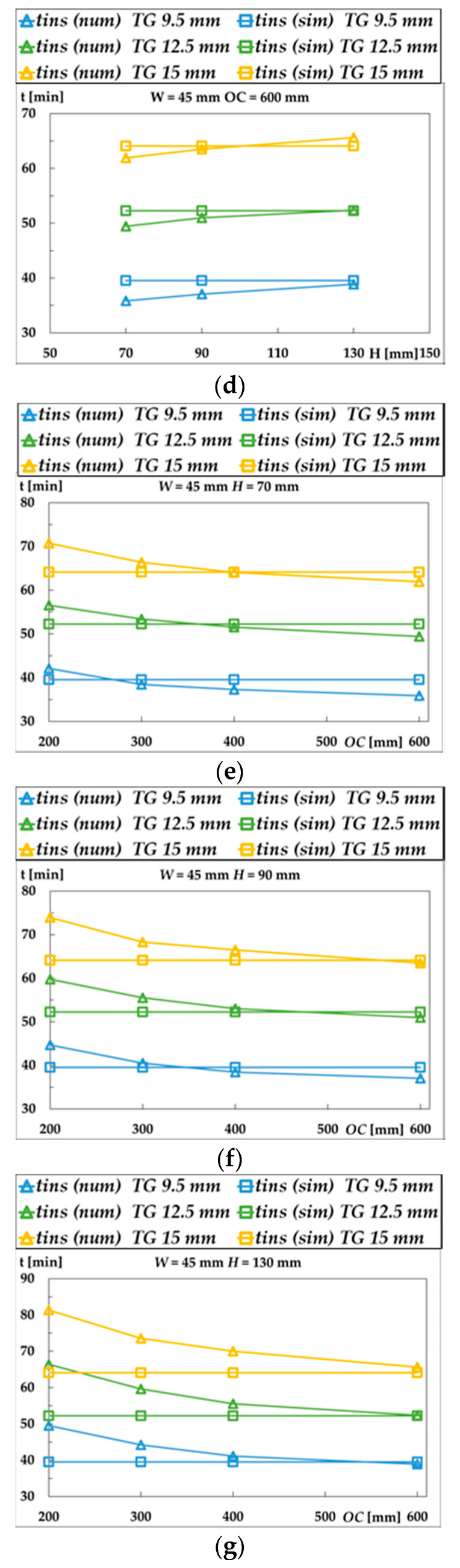
Comparison of wall fire-resistance time according to the simplified model (sim) and numerical (num) results: (**a**) *OC* as 200 mm; (**b**) *OC* as 300 mm; (**c**) *OC* as 400 mm; (**d**) *OC* as 600 mm; (**e**) *H* as 70 mm; (**f**) *H* as 90 mm; (**g**) *H* as 130 mm.

Figure 3 represents the fire resistance of the LTF walls, regarding the insulation (I) criterion, when considering the variation of *H* and *OC*. The fire resistance increases with the depth of the studs, *H*, and reduces with the distance between them (*OC*), for every gypsum layer thickness *TG*. The fire resistance determined by the simplified model is not able to predict these variations.

**Figure 4:**
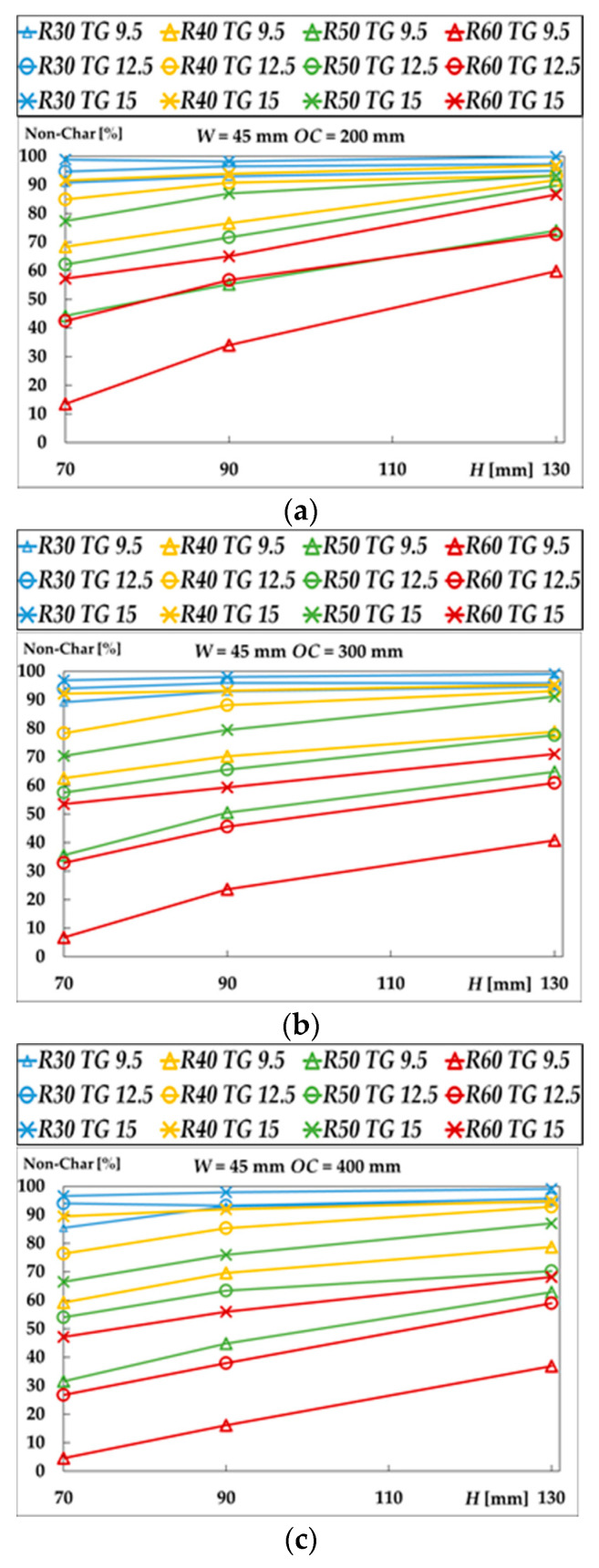

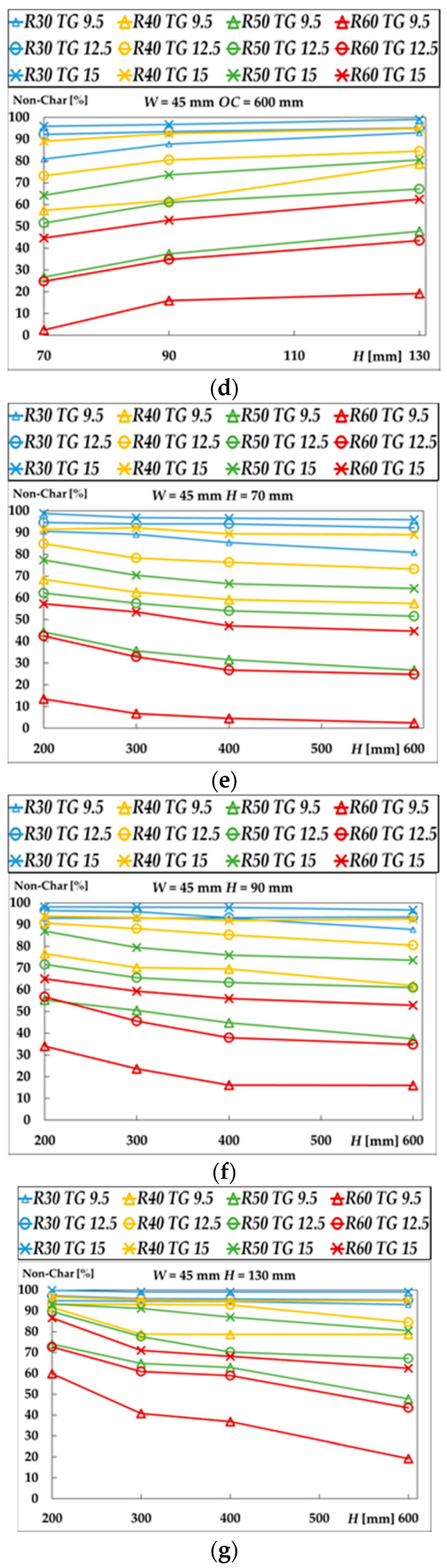
Evolution of the residual area (*RA*) of the central stud for 30, 40, 50 and 60 min: (**a**) *OC* as 200 mm; (**b**) *OC* as 300 mm; (**c**) *OC* as 400 mm; (**d**) *OC* as 600 mm; (**e**) *H* as 70 mm; (**f**) *H* as 90 mm; (**g**) *H* as 130 mm.

**Figure 5:**
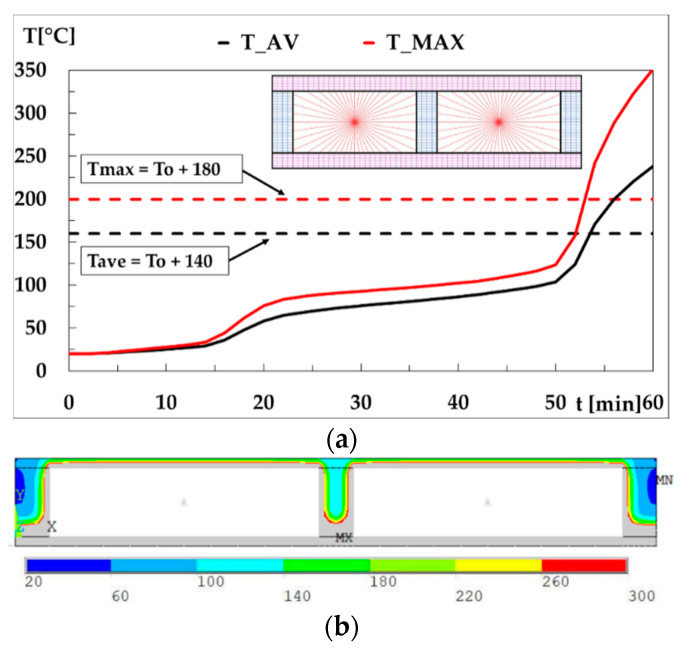
(**a**) Thermal performance of the specimen [90 × 45] + 12.5 × 1 + 400, time–temperature history for the unexposed side and finite element mesh; (**b**) Temperature for 50 min of the specimen.

On the other hand, Figure 4 represents the residual area (*RA*), or noncharred area, of the central stud after being submitted to fire periods of 30, 40, 50, and 60 min. The residual area increases with the dimension *H* and decreases with *OC*. Both the fire resistance time for the [90 × 45] + 12.5 × 1 + 400 case and the noncharred area of the central stud after 50 min of fire exposure are shown in Figure 5.

## 4. Discussion

From Figure 3, it can be seen that the separating function method gives the same fire resistance, independently of the depth of the stud. This is not the case with the numerical results, where an increase in the stud depth contributes to increasing the fire resistance. Thus, the variation of the cross-section depth (*H*) changes the fire resistance time of the LTF wall assembly insulation (I) of up to 11 min with respect to the separating function method. As the value of *H* increases, the area of the stud and of the cavity increases, resulting in a higher value of fire resistance of the insulation. The noncharred area (residual) depends on the variation of *H*. The residual stud area can vary from 1% to 38% for the most unfavourable cases.

From the numerical results presented in Figure 3, it can be also observed that the fire resistance decreases with the distance between studs (*OC*), with the numerical results (i) being higher than the ones obtained with the separating function method for low space distance and (ii) smaller for higher distances (*OC* = 600 mm). The variation of the *OC* can make a difference in the fire-resistance time of the LTF wall of up to 16 min when compared to the separating function method. An increase of 100 mm on the *OC* distance between studs may reduce the fire resistance of the LTF wall by 5 min, especially when considering a variation from 200 to 300 mm. This reduction will decrease by 2.5 min for larger spacings. It should be noted that the commonly used distances of *OC* are 400 and 600 mm, therefore, the difference between both methods is smaller in these cases. Despite this, there are some results where the separating function method [5] provides a fire resistance lower than the one obtained in the simulation, which is convenient for safety reasons but inconvenient regarding the economy. In contrast, other results show that the separating function method [5] provides a higher value of fire resistance in comparison to simulation results, which can be considered unsafe.

From Figure 4, it could be concluded that the residual area decreases with the distance between the studs. Therefore, the residual area is also affected by this geometry variation. By increasing the *OC* value from 200 to 600 mm, the residual stud area can vary from 0.7 to 41%, for the most unfavourable cases. In addition, according to Figure 4, it is also deduced that the residual area decreases with the depth of the stud, for any protection level (*TG*).

The biggest difference in the fire-resistance time of the insulation between the separating function method and the numerical results is 27%.

On average, the increase in fire resistance of insulation is 5 ± 0.4 min for each millimetre of gypsum thickness. This result is only valid for the currently used geometries and for the thickness of the gypsum layer (*TG*).

Thus, this study reveals the shortcomings of the simplified model used to determine the fire resistance in EN 1995-1-2 [6], because it does not consider the influence of the distance *OC* and depth *H* of the studs in the insulations fire resistance of LTF wall assemblies.

## 5. Conclusions

The contributions generated by this communication, related to the study of LTF assemblies against fire resistance, are the following: 

An increase in the distance between the studs (*OC*) causes a small influence of the studs on the fire resistance of the structure. The fire resistance and the percentage of noncharred areas are reduced.

An increase in the cross-sectional thickness of the studs (*H*) results in a higher cavity thickness between both sides of the wall, which causes an increase in the fire resistance and a higher percentage of noncharred area.

Although the simplified model presented by Frangi et al. (2010) [5], included in the new generation of Eurocode EN 1995-1-2 (2020) [6], provides much more similar results to the numerical model, there is a considerable difference when not taking into account the depth of the stud, *H*, and the distance between them, *OC.*

Thus, the results obtained represent an important contribution in the study of LTF assemblies against fire resistance. This investigation shows the relevant effect of the distance *OC* and depth *H* on the fire resistance of these LTF wall assemblies. Furthermore, it demonstrates that the separating function method [5], which is the one proposed to be used by practitioners, might be updated considering the effect of these parameter values (*OC* and *H*) on increasing its accuracy.

## Figures and Tables

**Table 1:** The fire resistance of the light timber-framed walls and the residual area of the central.

[H × W] + TG + OC	AREA [mm^2^]	*tins* (num) [min]	*tins* (sim) [min]	% Error	AREA R30 [%]	AREA R40 [%]	AREA R50 [%]	AREA R60 [%]
[70 × 45] + 9.5 × 1 + 200	3150	42.09	39.54	6.45	90.67	68.36	44.22	21.66
[90 × 45] + 9.5 × 1 + 200	4050	44.66	39.54	12.95	92.91	76.64	55.26	34.04
[130 × 45] + 9.5 × 1 + 200	5850	49.54	39.54	25.30	94.89	91.64	73.93	59.82
[70 × 45] + 12.5 × 1 + 200	3150	56.54	52.27	8.17	94.61	84.91	62.17	42.42
[90 × 45] + 12.5 × 1 + 200	4050	59.78	52.27	14.37	96.42	90.77	71.63	56.77
[130 × 45] + 12.5 × 1 + 200	5850	66.43	52.27	27.10	97.20	93.15	89.70	72.69
[70 × 45] + 15 × 1 + 200	3150	70.73	64.10	10.34	98.78	91.49	77.31	57.21
[90 × 45] + 15 × 1 + 200	4050	73.96	64.10	15.39	98.18	93.79	86.92	65.02
[130 × 45] + 15 × 1 + 200	5850	81.35	64.10	26.92	99.77	96.70	93.23	86.63
[70 × 45] + 9.5 × 1 + 300	3150	38.47	39.54	2.70	89.12	62.55	35.56	13.45
[90 × 45] + 9.5 × 1 + 300	4050	40.51	39.54	2.45	91.96	70.18	50.55	23.64
[130 × 45] + 9.5 × 1 + 300	5850	44.25	39.54	11.92	93.66	77.84	64.81	40.71
[70 × 45] + 12.5 × 1 + 300	3150	53.43	52.27	2.21	93.92	78.33	57.51	32.87
[90 × 45] + 12.5 × 1 + 300	4050	55.50	52.27	6.18	95.92	88.07	65.61	45.61
[130 × 45] + 12.5 × 1 + 300	5850	59.64	52.27	14.09	95.82	93.19	77.65	60.93
[70 × 45] + 15 × 1 + 300	3150	66.35	64.10	3.51	96.81	92.13	70.39	53.54
[90 × 45] + 15 × 1 + 300	4050	68.32	64.10	6.58	96.96	93.22	79.48	59.27
[130 × 45] + 15 × 1 + 300	5850	73.55	64.10	14.75	99.05	95.32	91.16	71.06
[70 × 45] + 9.5 × 1 + 400	3150	37.33	39.54	5.60	85.45	59.22	31.59	10.40
[90 × 45] + 9.5 × 1 + 400	4050	38.46	39.54	2.74	92.93	69.57	44.84	16.15
[130 × 45] + 9.5 × 1 + 400	5850	41.12	39.54	3.99	94.47	78.13	62.88	36.87
[70 × 45] + 12.5 × 1 + 400	3150	51.55	52.27	1.39	94.01	76.36	53.98	26.78
[90 × 45] + 12.5 × 1 + 400	4050	53.01	52.27	1.41	93.14	85.28	63.36	37.90
[130 × 45] + 12.5 × 1 + 400	5850	55.56	52.27	6.29	95.67	92.77	70.18	58.89
[70 × 45] + 15 × 1 + 400	3150	64.09	64.10	0.02	96.55	89.38	66.48	47.19
[90 × 45] + 15 × 1 + 400	4050	66.48	64.10	3.72	97.85	91.93	75.91	55.94
[130 × 45] + 15 × 1 + 400	5850	70.00	64.10	9.20	98.87	94.78	87.00	68.10
[70 × 45] + 9.5 × 1 + 600	3150	35.86	39.54	9.31	80.89	57.36	26.81	14.90
[90 × 45] + 9.5 × 1 + 600	4050	37.05	39.54	6.29	87.80	61.93	37.35	16.00
[130 × 45] + 9.5 × 1 + 600	5850	38.88	39.54	1.66	92.98	78.69	47.75	19.18
[70 × 45] + 12.5 × 1 + 600	3150	49.44	52.27	5.42	92.19	73.20	51.58	24.76
[90 × 45] + 12.5 × 1 + 600	4050	50.97	52.27	2.48	93.42	80.46	61.01	34.86
[130 × 45] + 12.5 × 1 + 600	5850	52.37	52.27	0.19	95.11	84.47	67.11	43.56
[70 × 45] + 15 × 1 + 600	3150	61.93	64.10	3.39	95.97	89.02	64.26	44.65
[90 × 45] + 15 × 1 + 600	4050	63.50	64.10	0.94	96.64	92.57	73.65	52.86
[130 × 45] + 15 × 1 + 600	5850	65.64	64.10	2.40	99.02	94.94	80.49	62.45

## Data Availability

Not applicable.
